# A minimum-error, energy-constrained neural encoder predicts an instantaneous spike-rate code

**DOI:** 10.1186/1471-2202-16-S1-P201

**Published:** 2015-12-18

**Authors:** Erik C Johnson, Douglas L Jones, Rama Ratnam

**Affiliations:** 1 Department of Electrical & Computer Engineering, University of Illinois, Urbana, IL 61801, USA; 2 Beckman Institute for Advanced Science and Technology, University of Illinois, Urbana, IL, 61801, USA; 3 Coordinated Science Laboratory, University of Illinois, Urbana, IL, 61801, USA; 4 Advanced Digital Sciences Center, Illinois at Singapore Pte. Ltd., Singapore

## 

**Figure 1:**
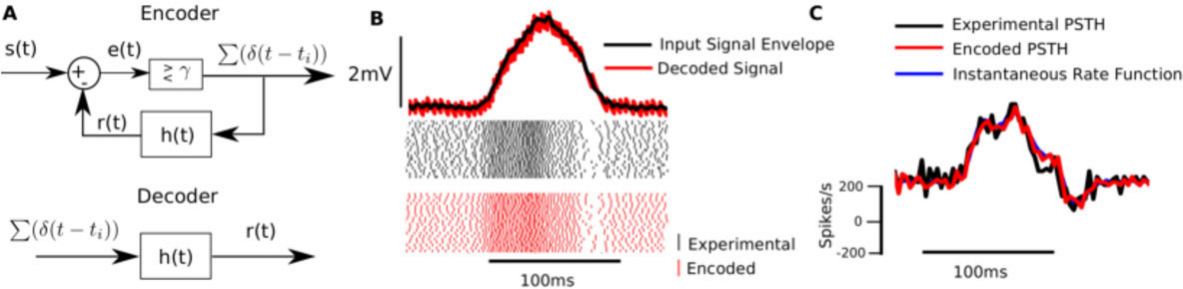
**A. Schematic of energy-constrained, minimum-error encoding model**. **B**. A perturbation of the fish's electric field (top) and the response of primary electrosensory neuron (below, experimental and optimal coder). Model produces *r*(*t*) in close match with *s*(*t*) and optimal spike-times. **C**. Smoothed PSTH of the spike trains, and the predicted instantaneous rate function (blue, obscured).

An action potential (spike) is metabolically expensive to generate, and it is likely that selective pressure has been exerted on the nervous system to generate energy-efficient neural codes [1]. An additional constraint in sensory systems is that the encoding should also represent stimuli with minimal error. We postulate that this leads to a trade-off between energy expenditure and encoding error, and propose that an optimal neural code should minimize encoding error subject to a constraint on the energy expended. A first approximation of energy expenditure by a spiking neuron is *E *= *b *+ *kN_s_*, where *E: *expended energy, *b: *baseline rate, *k: *cost per spike, and *N_s_*: the number of spikes fired. Figure 1A depicts the encoding of an input signal *s*(*t*) as a spike-train. Given an energy constraint *E*, the goal is to obtain the best possible reconstruction *r*(*t*) of *s*(*t*), with a simple low-pass filter *h*(*t*) = *A *exp(-*t*/τ) (mimicking a post-synaptic cell membrane) such that average squared reconstruction error *e*(*t*) = *s*(*t*) - *r*(*t*) (Figure 1A) is minimized. Previously we showed that minimal error encoding, subject to a spike-rate constraint, is equivalent to a non-resetting dynamic threshold spike-firing model [2] with an dynamic threshold *h*(*t*) and firing level *γ*. Here, we show that this encoder can be interpreted as an instantaneous rate encoder with rate *i*(*t*) = (*s*(*t*)/*τ *+ *s'*(*t*))/*A*. The function closely approximates the PSTH. We tested the instantaneous rate coder by predicting: i) spike-times (Figure 1B, see raster), and reconstruction (Figure 1B, top panel), and ii) the smoothed PSTH (Figure 1C) for single-neuron data from p-type primary electrosensory neurons of a weakly-electric fish (*Apteronotus leptorhynchus)*. For signals with little variability, this predicts a rate-code of signal intensity. For signals with high variability, the spike-rate is driven by the changes in the signal (i.e., its derivative). We conclude that optimal encoder can optimally time spikes while maintaining high coding fidelity, which can be interpreted as an instantaneous rate code.
